# Exogenous Cytokine-Free Differentiation of Human Pluripotent Stem Cells into Classical Brown Adipocytes

**DOI:** 10.3390/cells8040373

**Published:** 2019-04-24

**Authors:** Masako Oka, Norihiko Kobayashi, Kazunori Matsumura, Miwako Nishio, Kumiko Saeki

**Affiliations:** Department of Disease Control, Research Institute, National Center for Global Health and Medicine, Tokyo 162-8655, Japan; moka@ri.ncgm.go.jp (M.O.); nkobayashi@ri.ncgm.go.jp (N.K.); kmatsumura@ri.ncgm.go.jp (K.M.); miwako-nishio@ri.ncgm.go.jp (M.N.)

**Keywords:** cytokine-free, brown adipocyte, human pluripotent stem cells, size-controlled spheroids

## Abstract

We previously established a method for a directed differentiation of human pluripotent stem cells into classical brown adipocytes (BA) by forming aggregates via massive floating culture in the presence of a specific cytokine cocktail. However, use of recombinant cytokines requires significant cost. Moreover, an enforced differentiation by exogenously added cytokines may amend skewed differentiation propensity of patient’s pluripotent stem cells, providing unsatisfactory disease models. Therefore, an exogenous cytokine-free method, where cytokines required for differentiation are provided in an auto/paracrine manner mimicking natural developmental process, is beneficial. Here we show that, if human pluripotent stem cells are cultured as size-controlled spheroids (100–120 µm radius, 2000–2500 cells/spheroid) in a mutually segregated manner with half-change of the medium every other day, they differentiate into classical BA via an authentic MYF5-positive myoblast route in the absence of exogenous cytokines. Differentiated BA exerted thermogenic activity in transplanted mice in response to beta-adrenergic receptor agonist stimuli. The cytokine-free differentiation method has further advantages in exploring BATokines, BA-derived physiologically active substances. Indeed, we have found that BA produces an unknown small (<1000 Da), highly hydrophilic molecule that augments insulin secretion from pancreatic beta cells. Our upgraded technique will contribute to an advancement of stem cell study for diverse purposes.

## 1. Introduction

Human pluripotent stem cells, which include human embryonic stem cells (hESC) and human-induced pluripotent stem cells (hiPSC), are expected as a powerful tool for regenerative medicine. Several clinical trials are currently in progress [1]. For further enhancement of hESC/hiPSC-based regenerative medicine, however, cost issue needs to be addressed. For example, supplementation of the differentiation medium with multiple kinds of expensive recombinant cytokines raises economic hurdles. An effort to eliminate the need for supplying synthetic cytokines to the differentiation medium has been started. A method for a cytokine-free directed differentiation of human pluripotent stem cells into hemogenic endothelium was reported, where a low molecular weight compound, a Wnt agonist, was used in replacement of the recombinant cytokines [2]. Although successful cases are currently limited, it is theoretically possible to eliminate the requirement of exogenous cytokines if endogenous counterparts are properly expressed to serve as auto/paracrine factors for the differentiation to reproduce natural developmental process. Removal of exogenous cytokines from the differentiation system has an additional merit that it can eliminate the risk of adverse effects of recombinant cytokines, which might possibly be brought into living body together with the transplanted cells. This is especially important when transplantation therapies for non-malignant diseases, such as morbid obesity, are considered. It is known that transplantation of brown adipose tissue (BAT) exerts therapeutic effects for obesity and obesity-related metabolic disorders in mice [3]. Transplantation of hESC/hiPSC-derived brown adipocyte (BA) may provide a new option for the treatment of morbid obesity if exogenous cytokine-free method for the differentiation is established.

We previously reported a method for a directed differentiation of hESC/hiPSC into classical brown adipocyte (BA) [4,5]. Classical BA is a unique fat cell that produces heat via uncoupled respiration at mitochondria. It has abundant large-size mitochondria, which contain ladder-like dense cristae. It is derived from Myf5-positive myoblasts [6], which are originated from a dorsocentral portion of the somite. In contrast to MyoD-positive myoblasts, which give rise to trunk and limb muscles, Myf5-positive myoblasts give rise to back muscles, which are known as anti-gravity muscles with the most abundant mitochondria [7].

Compared to white adipose tissues (WATs) such as subcutaneous and visceral fat depots, classical brown adipose tissues (BAT), which we call here BAT for simplicity, are small in quantity. The value of “BAT weight (g)/body weight (kg)” is calculated as lower than 2 g/kg (i.e., 150 g/human body) [8] if the average body weight of adult humans is estimated as 75 kg. On the other hand, it is estimated as 5 g/kg in murine cases (i.e., 150 mg/30 g) [9]. They locate at specific sites of the body including intrascapular spaces (mice and newborn humans), cervical, axillary, and paravertebral regions (mice and humans) [10,11,12,13,14]. There is no gender difference in the detection efficiency of BAT by positron emission tomography in combination with computed tomography (PET-CT) examinations in humans [12]. During thermogenesis, BA burns triglyceride stored in its multilocular lipid droplets. Ever since the discovery of BAT in adult humans in 2009 [11,15,16,17], BA has been attracting attention as a promising target of drug discovery for the treatment of obesity and diabetes. It was shown that transplantation of BAT ameliorated obesity and improved glucose metabolism in high-fat diet-fed mice [3]. Although similar roles for BAT have been suggested in humans [18], the research of human BAT has been lagging because it is difficult to obtain sufficient amounts of clinical samples due to ethical reasons. In addition, BA is incompetent for cryopreservation and expansion in vitro. Therefore, hESC/hiPSC are expected as an ideal tool for stable provision of human BA.

In our previous method, we used hESCs/hiPSCs that were maintained on mouse embryonic fibroblast (MEF) layer as crops of cells. After detachment from MEF layer, hESC/hiPSC crops were directly transferred to a low attachment plate without dissociation. By this process, hESC/hiPSC crops were turned into cell aggregates. We cultured these aggregates in the presence of IL-6, VEGFA, KITLG, FLT3LG, and BMP4 for eight days with half-change of the medium every other day (Step 1). At Day 8, the aggregates were transferred onto gelatin-coated plates and cultured for additional two days in the presence of IL-6, VEGFA, KITLG, FLT3LG, and BMP7 (Step 2). BMP4 is required for cell aggregate formation per se, whereas IL-6, VEGFA, KITLG, and FLT3LG contribute to the process of lipid droplet formation [4]. IL-6 is also known to be required for executing metabolism-improving actions of BA [17]. BMP7 supplementation in Step 2 enhanced BA differentiation [4,5] in parallel with the report that BMP7 knockout mice showed considerably small BAT [19], suggesting that BMP7 is required for the expansion of BA progenitors. However, supplementation of recombinant BMP7 was not a requisite for BA differentiation [4] since the expression of BMP7 was induced during Step 1. BMP4 is required for generation of spheroid per se, IL-6 is required for functional maturation of BA for metabolism regulation; other cytokines seem to be involved in at least multilocular lipid droplet formation. Therefore, if an ideal microenvironment for BA differentiation is prepared for human pluripotent stem cells, other cytokines can probably be removed as well. In our earlier work, to establish a method for cytokine-free maintenance of hESCs [20], we showed that strict control of the size and the density of hESC crops was critical to eliminate the need of exogenous cytokines. Therefore, we searched the optimal cell handling condition, focusing on the size and the density of the aggregates, to establish a cytokine-free method for the differentiation of human pluripotent stem cells into BA.

Here, we show that hESCs/hiPSCs spontaneously differentiate into BA in the absence of a cytokine cocktail, if: (1) Size-controlled spheroids of 100–120 μm radiuses are created from completely dissociated hESCs/hiPSCs and (2) each spheroid is cultured independently in a microwell under a condition where a half volume of the medium was changed every other day. Spheroids must be cultured in a mutually segregated fashion, because fusing and clumping of the spheroids lowered the differentiation efficiency. Expressions of the endogenous genes that code the components of a cytokine cocktail were induced during the differentiation process, suggesting that these cytokines work as auto/paracrine factors for the differentiation into BA. Although the molecular mechanism for cytokine-free differentiation of hESCs/hiPSCs into BA remains elusive, our new technique will contribute to an acceleration of BA-based regenerative medicine for the treatment of metabolism disorder and the promotion of the exploration of BA-derived physiologically active substances, which are called BATokines.

## 2. Materials and Methods

### 2.1. Immature Maintenance of Human Pluripotent Stem Cells

Human embryonic stem cell (hESC) lines, KhES-1 and KhES-3, were provided by the Institute for Frontier Life and Medical Sciences, Kyoto University [21] and used under approval of Human Embryonic Stem Cell Research Ethical Review Board of National Center for Global Health and Medicine (NCGM) with acceptance of Ministry of Education, Culture, Sports, Science and Technology of Japan. SeV-iPS(BJ) [4], a Sendai virus vector-based human iPS cell (hiPSC) line, was established from BJ human newborn foreskin fibroblast line (CRL-2522^TM^; ATCC, Manassas, VA, USA) by using iPS-Tune^TM^ (ID Pharma Co., Ltd., Ibaraki, Japan) [4]. 201B7, a retroviral vector-based hiPSC line, was provided by CiRA at Kyoto University [22]. hESCs/hiPSCs were maintained by a feeder-free system using 4 mL StemFit^TM^ AK02N (Ajinomoto Healthy Supply Co., Inc., Tokyo, Japan) on a 60-mm diameter plate pre-coated with 1:100 diluted Vitronectin (VTN-N) Recombinant Human Protein, Truncated, A14700 (Thermo Fisher Scientific Inc., Waltham, MA, USA) or iMatrix (10 µL/60 mm plate) (Nippi. Inc., Tokyo, Japan). Medium was refreshed every other day. When cell densities reached 70%–80% confluency, hESCs/iPSCs were harvested by a treatment with 1 mL TrypLE^TM^ Express (Thermo Fisher Scientific Inc., Waltham, MA, USA) for 5–10 min at 37 °C, followed by mild pipetting using a 1000 µL tip. After washing with PBS, cells were seeded onto a new 60-mm diameter pre-coated plate (0.5–1 × 10^5^ cells/plate) using 4 mL StemFit^TM^ AK02N (Ajinomoto Healthy Supply Co.) supplemented with 40 µL RevitaCell supplement (Thermo Fisher Scientific Inc.), a ROCK inhibitor. Proliferation rate of the cells was 2.0 logs in 5–7 days.

### 2.2. Differentiation into Classical BA

hECSs/hiPSCs were harvested and dissociated into single cell by treatments with TrypLE^TM^ Express (Thermo Fisher Scientific Inc.) and suspended in the differentiation medium supplemented with RevitaCell supplement (Thermo Fisher Scientific Inc., Waltham, MA, USA). Size-controlled spheres were generated by transferring 200 μL aliquot of cell suspension into each well of Elplasia^®^ 3D discovery tool (RB 500 400 NA 96; Kuraray Co. Ltd., Tokyo, Japan), which has 90 microwells per well, at the density of 1.8–2.2 × 10^5^ cells/well (2000–2500 cells/spheroid). Cells were cultured at 37 °C in CO_2_ incubator (5% CO_2_) for 8 days, during which a half volume of the differentiation medium (i.e., 100 µL) was refreshed every other day (Step 1). Then, spheroids were transferred onto a 60 mm type I collagen-coated plate (4010-010-MYP, AGC Techno Glass Co., Ltd., Shizuoka, Japan) and cultured in the differentiation medium for 2 days (Step 2). In some pilot studies, a 96-well V-shaped low attachment plate (PrimeSurface^TM^ 96V well culture plate, Sumitomo Bakelite Co., Ltd. Tokyo, Japan) was used for spheroid formation (Step 1) and a 60 mm gelatin-coated plate was used for adherent culture (Step 2) as previously described [4,5] (Supplementary Figure S1).

The composition of the differentiation medium in *cytokine-free* method is as follows: 1:1 ratio of IMDM and Ham’s F12, 1:100 synthetic lipids, 1:100 ITS-A, 2 mM GlutaMAX™ II (Gibco #35050-061, Thermo Fisher Scientific Inc., Waltham, MA, USA), 50 µg/mL ascorbic acid-2-phosphate. In addition to the cytokine cocktail, BSA, α-MTG, and PFHMII are deleted from the original medium.

The composition of the original differentiation medium [4,5] was as follows: 1:1 ratio of IMDM (I3390, Sigma Chemical Co. LLC, St. Louis, MO, USA) and Ham’s F12 (087-08335, FUJIFILM WAKO Pure Chemical Industries, Osaka, Japan), 5 mg/mL bovine serum albumin (BSA) (A802, Sigma Chemical Co. LLC), 1:100 synthetic lipids (Gibco # 11905-031, Thermo Fisher Scientific Inc.), 450 μM α-monothioglycerol (α-MTG) (207-09232, WAKO Pure Chemical Industries), 1:100 insulin-transferrin-selenium (ITS-A, Thermo Fisher Scientific Inc.), 2 mM GlutaMAX™ II (Gibco #35050-061, Thermo Fisher Scientific Inc.), 5% protein free hybridoma mix (PFHMII, Gibco #12040-077) (Thermo Fisher Scientific Inc.), 50 µg/mL ascorbic acid-2-phosphate (A-8960, Sigma Chemical Co. LLC), 20 ng/mL BMP4, 5 ng/mL VEGFA, 20 ng/mL KITLG, 2.5 ng/mL FLT3LG, 2.5 ng/mL IL-6, and 5 ng/mL IGF-II for Step1. For Step 2, 10 ng/mL BMP7 was used instead of 20 ng/mL BMP4.

### 2.3. Live Cell Staining of Mitochondria and Lipid Droplets

Cells were incubated at 37 °C in CO_2_ incubator (5% CO_2_) for an hour in the presence of 100 nmol/l MitoBright Green (Dojindo Molecular Technologies, Inc., Kumamoto, Japan), 1 µmol/l Lipi-Red (Dojindo Molecular Technologies), and 130 µg/L Hoechst 33342 (Dojindo Molecular Technologies), which are live cell imaging probes for mitochondria, lipid droplets, and nuclei, respectively. In some experiments, Lipi-Green (Dojindo Molecular Technologies) was used for the detection of lipid droplets. Stained cells were observed under BZ-X710 All-in-One fluorescence microscope (KEYENCE Corp., Osaka, Japan) by a phase contrast mode or a fluorescence mode.

### 2.4. Immunostaining and TUNEL Assays

For immunostaining, differentiated cells were fixed by cold Acetone/Methanol (1:1). The 1st antibody reaction was performed by using either a rabbit polyclonal anti-human UCP1 antibody (ab10983; Abcam, Cambridge, UK) or a normal IgG (sc-2027; Santa Cruz Biotechnology, Dallas, TX, USA), and the 2nd antibody reaction was performed by using an Alexa Fluor^®^ 568-conjugated goat anti-rabbit IgG antibody (A11036; Thermo Fisher Scientific Inc.). To evaluate the viability of spheroids, TUNEL assays were performed. Spheroids were fixed by using 1% paraformaldehyde in a buffered solution and embedded in paraffin blocks. Spheroid slices were subjected to TUNEL assays, which were performed by using MEBSTAIN Apoptosis TUNEL Kit Direct (8445, Medical and Biological Laboratories Co. Ltd., Nagoya, Japan) according to the manufacturer’s instructions.

The samples were observed either under BZ-X710 All-in-One fluorescence microscope (KEYENCE Corp) or a confocal laser scanning microscope FLUOVIEW FV1000 (Olympus Corp., Tokyo, Japan).

### 2.5. Quantitative Reverse Transcription Polymerase Chain Reaction (qRT-PCR)

Total RNA was extracted from 1.5–2.0 × 10^6^ cells using RNeasy Mini Kit (QIAGEN, Hilden, Germany) along with DNase I treatment according to the manufacture’s guidance. cDNA was prepared from 2.5 µg RNA via RT reaction in 20 µL solution using SuperScript IV VILO (Thermo Fisher Scientific Inc.). qPCR was performed by applying 2 µL of 1/10-diluted cDNA template to StepOnePlus^TM^ real time PCR System (Thermo Fisher Scientific Inc). The nucleotide sequences of the primers used in qPCR were as follows: PRDM16, forward: CGAGGCCCCTGTCTACATTC, reverse: GCTCCCATCCGAAGTCTGTC; PPARG, forward: AGCCTGCGAAAGCCTTTTGGTG, reverse: GGCTTCACATTCAGCAAACCTGG; UCP1, forward: AAATCAGCTCCGCCTCTCTC, reverse: TGCCACTCCTCCAGTCGTTA; MYF5, forward: GATGGCATGCCCGAATGTAAC, reverse: GCAATCCAAGCTGGATAAGGA; BMP7, forward: GGGCTTCTCCTACCCCTACA, reverse: TGTTCCACGAGGTTGACGAA; BMP4, forward: AAAGTCGCCGAGATTCAGGG, reverse: GACGGCACTCTTGCTAGGC; KITLG, forward: AATCCTCTCGTCAAAACTGAAGG, reverse: CCATCTCGCTTATCCAACAATGA; FLT3LG, forward: AAAATCCGTGAGCTGTCTGAC, reverse: TGACAAAGTGTATCTCCGTGTTC; VEGFA, forward: AGGGCAGAATCATCACGAAGT, reverse: AGGGTCTCGATTGGATGGCA; IL-6, forward: ACTCACCTCTTCAGAACGAATTG, reverse: CCATCTTTGGAAGGTTCAGGTTG; GAPDH, forward: CCACTCCTCCACCTTTGAC, reverse: ACCCTGTTGCTGTAGCCA.

### 2.6. In Vivo Calorigenic Analyses

hESC/hiPSC-derived BAs were subcutaneously transplanted and the calorigenic activity was evaluated by using an infrared camera as previously described [4,5] with following modifications. BAs were washed by prechilled PBS and transferred into prechilled 1.5 mL tube, where 30 µL prechilled fibrin gel was added and mixed. The 100 µL of fibrin gel was prepared by mixing chilled 89.3 µL aqueous solution (2.8 mg/mL) of fibrinogen from bovine plasma type I-S (F8630, Sigma Chemical Co. LLC), prechilled 6.7 µL collagen type I rat tail (354236, Corning Inc. One Riverfront Plaza, Corning, NY, USA), and prechilled 3 µL aprotinin from bovine lung saline solution (A6279, Sigma Chemical Co. LLC), which was successively added by prechilled 1 µL of 50 U/mL thrombin from bovine plasma lyophilized powder (T4648, Sigma Chemical Co. LLC). The 30 µL prechilled fibrin gel or BA-embedded fibrin gel was subcutaneously transplanted in the gluteal region of 5–6 week-old male ICR mice or NOD/ShiJic-*scid* (NOD/SCID) mice (CLEA Japan, Inc., Tokyo, Japan), whose hair was removed in broad areas around hip using hair removal cream 3 days before, through a 5 mm incised part using a 1000 µL pipette tip. After 48 to 96 h (for short-term assays) or 9 days (for longer-term assays) from transplantation, mice were anesthetized by using a combination anesthetic (0.3 mg/kg of medetomidine, 4.0 mg/kg of midazolam, and 5.0 mg/kg of butorphanol). After 15 min, 30 µmol/kg of isoproterenol (I2760, Sigma Chemical Co. LLC) was administrated from the tail vein, and after another 4 to 6 min, dermal temperature was measured by Thermo GEAR G120/G100 (NEC Avio Infrared Technologies Co., Ltd., Tokyo, Japan). For cold acclimation, mice were kept at 5 °C for 96 h using KCLP-1000 II CT (Nippon Medical and Chemical Instruments Co. Ltd., Osaka, Japan). All animal care procedures were approved by the Animal Care and Use Committee of the Research Institute, National Center for Global Health and Medicine (NCGM) (Authorization No. 18018, 18019), and complied with the procedures of the Guide for the Care and Use of Laboratory Animals of NCGM.

### 2.7. Preparation of the Supernatant of BA (BA-SUP) and an Evaluation of its Biological Activity

Regarding BAs generated by a conventional method, BA-SUP was prepared as follows. The medium of Day 10 BA, which was cultured on a gelatin-coated 60 mm plate, was replaced by 2 mL HEPES-based Krebs-Ringer (KR) buffer supplemented with 16.8 mM glucose. The composition of HEPES-based KR buffer is as follows: 128 mM NaCl, 5 mM KCl, 2.7 mM CaCl_2_, 1.2 mM MgSO_4_, 1 mM Na_2_HPO_4_, and 20 mM HEPES (pH 7.4). After BA was cultured in HEPES-based KR buffer supplemented by 16.8 mM glucose in a CO_2_ incubator (5% CO_2_) at 37 °C for 16 h, the supernatant was collected. For control, HEPES-based KR buffer supplemented by 16.8 mM glucose was used. To evaluate in vivo effects, BA-SUP was subcutaneously injected (12.5 µL/g) into 10-week-old male ICR mice (*N* = 6). After 16 h fasting, blood samples were obtained from tail vein and blood glucose levels, serum insulin concentrations and blood triglyceride values were measured as previously described [4]. The BA-SUP prepared by this procedure was used for insulin secretion assay using MIN6 cells (Figure 8b).

Regarding BAs generated by a cytokine-free method, BA-SUP was prepared as follows. The medium of BA, which was cultured on a type 1 collagen-coated 60 mm plate, was replaced by 1.5 mL TRIS-based KR buffer supplemented by 2.8 mM glucose. The composition of TRIS-based KR buffer is as follows: 119 mM NaCl, 4.74 mM KCl, 1.19 mM CaCl_2_, 1.19 mM MgCl_2_, 1.19 mM KH_2_PO_4_, 25 mM NaHCO_3_, and 10 mM TRIS (pH 7.4). After BA was cultured in a CO_2_ incubator (5% CO_2_) at 37 °C for 16 h, the supernatant was collected. For control, TRIS-based KR buffer supplemented by 2.8 mM glucose was used.

### 2.8. Insulin Secretion Assay In Vitro

MIN6 cells (clone 4) [23] were maintained using DMEM, high glucose (4.5 g/L glucose) supplemented with l-glutamine, β-mercaptoethanol, sodium bicarbonate, and heat inactivated feta calf serum [23,24]. Before assay, MIN6 was seeded on a 96-well multi-well plate at the density of 1.2 × 10^5^/well and cultured in a CO_2_ incubator (5% CO_2_) at 37 °C for 40–48 h. Then, the medium was replaced by KR buffer supplemented by low glucose (2.8 mM glucose) and cells were cultured for 2–3 h for glucose starvation. Then, the medium was replaced by 50 µL BA-SUP or control KR buffer. After incubation for another 2–3 h, the supernatant was collected, diluted by control buffer (1:10–1:50), and used as a template for New HTRF^®^ High Range Insulin Assay (Cisbio Bioassays, Codolet, France). In this assay, Europium cryptate-coupled anti-insulin antibody A (1 µL), XL665-coupled anti-insulin antibody B (2 µL), 5 µL template, and 72 µL dilution buffer were mixed (totally 80 µL) and incubated for 6 h at room temperature in a 96-well half-area plate (Greiner Bio-One, Kremsmunster, Austria). Fluorescence emissions were measured by a multi-microplate reader (Tristar2 LB 942 Berthhold technologies GmbH and Co. KG, Bad Wildbad, Germany). A 337 nm nitrogen laser was used for fluorophore excitation, and a 620 nm filter was used for Europium cryptate and 665 nm filter was for the XL665 fluorescence detection. The data were evaluated by the value of fluorescence ratio (665 nm/620 nm). Insulin concentration was calculated using a standard curve created by serially diluted (0.61–150 ng/mL) insulin solution (Insulin standard, 500 ng/mL; Cisbio Bioassays, Codolet, France).

### 2.9. Statistics

Experiments were performed in multiplicate both in vitro (*N* = 3–4) and in vivo (*N* = 6 mice) as comparison between two groups and the data were analyzed by *t*-test.

### 2.10. Partial Purification of a Novel Insulin Secretion-Stimulating Factor from BA-SUP by Using High-Pressure Liquid Chromatography (HPLC)

BA-SUP was desalted by using PD MidiTrap™ G-10 (GE Healthcare, Chicago, IL, USA). Before use, the column was equivalated by 10 mM Tris-HCl (pH8.0). After applying 1 mL BA-SUP, PD10 column was washed by 16 mL 10mM Tris-HCl (pH 8.0), and the sample was eluted by 1.2 mL 10 mM Tris-HCl (pH 8.0). The eluted samples were promptly frozen in liquid nitrogen and lyophilized by using a centrifugal concentrator Spin dryer light VC-36R (TAITEC Corp., Saitama, Japan). The dried samples were dissolved in 50 µL TRIS-based KR buffer, centrifuged with 10000 G for 10 min by refrigerated centrifuge MX-301 (TOMY SEIKO Co. Ltd., Tokyo, Japan), and applied to HPLC (Prominence Inert LC System, Shimadzu Corp., Kyoto, Japan) fitted with Superdex 75 5/150GL (GE Healthcare), a gel filtration column. TRIS-based KR buffer (2.8 mM glucose) was used as mobile phase with flow rate of 0.075 mL/min. Samples were collected every one minute, desalted, lyophilized, and dissolved in 50 µl TRIS-based KR buffer, each of which was subjected to the insulin secretion assay using MIN6 cells. Active fractions were mixed, desalted, lyophilized, dissolved in 50 µL 0.05% acetonitrile/0.1% acetic acid, and applied to reverse phase HPLC, which was fitted with Synergi 4 µm Fusion-RP 80A (Phenomenex Inc., Torrance, CA, USA), a C18, polar embedded column. Two kinds of mobile phase (solution A: 0.05% acetonitrile/0.1% acetic acid/H_2_O, Solution B: 90% acetonitrile/0.1% acetic acid/H_2_O) were run at the rate of 0.1 mL/min with the following program: 100% A (from 0 to 15 min), linear gradient of 100% A (at 15 min) to 100% B (at 30 min), 100% B (after 30 min). Samples were collected by each one minute until 8.5 min, then by every 2 min until 16 min. The collected samples were lyophilized, dissolved in 50 µL TRIS-based KR buffer, and subjected to the insulin secretion assay. Data on fractionation were analyzed by using LabSolutions version 5.93 software (Shimadzu Corp.).

### 2.11. Establishment of Knock-In hESC Line

To integrate 2A-TdTomato-fragment into the region upstream of the stop codon of *gapdh* gene, we used HITI method [25]. Each 30 µM of crRNAs, 5′-UCUAGGUAUGACAACGAAUU-3′ (for Genomic DNA digestion) and 5′-CAUGCGAGCACGAAUUAAUU-3′ (for vector digestion), were annealed with 30 µM Alt-R^®^ tracrRNA (Integrated DNA Technologies, Inc., Coralville, IA, USA) using nuclease-free duplex buffer according to the manufacture’s guidance (Integrated DNA Technologies, Inc.). The annealed products (1 µL for each) were mixed with 1 µL recombinant Cas9 protein (Alt-R^®^ S.p. Cas9 Nuclease V3; Integrated DNA Technologies, Inc.), 1.5 µL 1M KCl, 1 µL 0.2M HEPES buffer (pH 7.4), and water (up to 10 µL), and incubated for 10 min at room temperature. Then, 3 µg of a circular knock-in vector was added, and the mixture was transfected into 8 × 10^5^ hESCs (KhES-3) by using Nucleofector™ with human stem cell kit ver2 according the manufacture’s guidance (Lonza Group AG, Basel, Switzerland). Thereafter, cells were seeded onto iMatrix-coated 60 mm plate. The transfected cells were selected by puromycin resistance and the cloned knock-in lines were further confirmed their genomic DNA by performing PCR. Knock-in vector (GAPDH-2A-NLS-TdTomato-PuromycinR) was constructed in-house. TdTomato was obtained from pQC NLS TdTomato IX, which is a gift from Connie Cepko (Addgene plasmid #37347; http://n2t.net/addgene:37347; RRID: Addgene_37347).

## 3. Results

### 3.1. Adaptation of Human Pluripotent Stem Cells to a Feeder-Free Culture and an Induction of Differentiation

To refine our original method for a directed differentiation human pluripotent stem cells into classical BA [4,5] to a xeno-free and cell number-controlled system, the modes of cell handling were upgraded in two points: (1) Immature hESCs were adapted to a feeder-free culture system, and (2) cells were dissociated into single cell in each subculture and differentiation procedure. We tested several commercially available systems and found that they similarly supported self-renewal of immature human pluripotent stem cells if a ROCK inhibitor was used along with the systems. However, when dissociated cells were subjected to differentiation into BA, the efficiency of differentiation significantly varied among the systems. To induce differentiation into BA, we seeded dissociated cells in a 96-well V-shaped low attachment plate to generate spheroids (Supplementary Figure S1A). These spheroids were cultured in the differentiation medium supplemented by a cytokine cocktail (VEGFA, IL-6, KITLG, FLT3-LG, BMP4) for eight days with half-change of the medium every other day (Step 1), followed by two-day adherent culture in the differentiation medium supplemented by another cytokine cocktail (VEGFA, IL-6, KITLG, FLT3-LG, BMP7) (Step2) as we previously reported [4,5]. Among the systems we tested, StemFit^TM^ AK02N (Ajinomoto Healthy Supply Co., Inc., Tokyo, Japan) provided the most promising results (Supplementary Figure S1B). Therefore, in our upgraded method, we used human pluripotent stem cells that were maintained by StemFit^TM^ AK02N (Ajinomoto Healthy Supply Co.), adding a ROCK inhibitor after every cell-dissociating procedure.

### 3.2. Culture of Size-Controlled Spheroids in Microwells Eliminates the Need for Exogenous Cytokines

**Figure 1:**
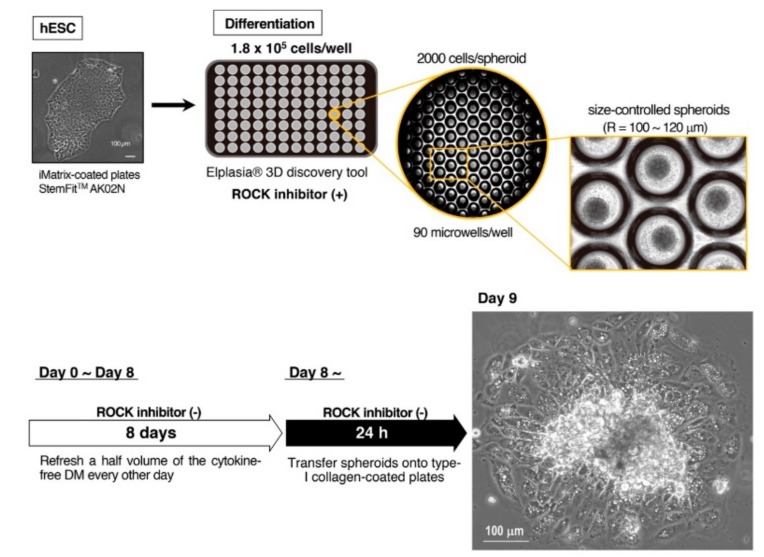
Generation of brown adipocytes (BA) from size-controlled human embryonic stem cells (hESC) spheroids using a cytokine-free differentiation medium. Feeder-free hESCs (KhES-3) maintained by StemFit^TM^ AK02N (Ajinomoto Healthy Supply Co.) on iMatrix-coated plates were dissociated into single cell. When cells were seeded at the density of 1.8 × 10^5^ cells/well on Elplasia^®^ 3D discovery tool (Kuraray Co. Ltd.) using cytokine-free differentiation medium in the presence of a ROCK inhibitor, spheroids with 100–120 µm radiuses were generated. A half volume of the medium was replaced by a fresh cytokine-free differentiation medium without a ROCK inhibitor every other day until day 8, when cells were transferred onto type 1 collagen-coated plates. at day 9, cells showed multiple lipid droplets and abundant mitochondria in their cytosol as observed under phase contrast microscopy (right lower panel). Although the central region of the attached spheroids was not always completely expanded, they were positively stained by Oil Red O (data not shown) as in the case of our original method [4].

Applying our upgraded method, we planned to examine the role for each cytokine by deleting it from the differentiation medium. Before performing this examination, however, we made an additional refinement in the spheroid-generating step. After testing several commercially available tools to generate size-controlled spheroids, we found that Elplasia^®^ 3D discovery tool (Kuraray Co. Ltd., Tokyo, Japan) was most fitted for our purpose because routine half-change of the medium was accomplished with the least labors and lowest cell damages. This tool has 90 smooth round microwells in each well (Figure 1, upper) to save spheroids from fusion.

We first determined the optimal number for cell seeding by examining the relationship between the number of seeded cells and the size of spheroids. We found that 1.8–2.2 × 10^5^ cells/well (i.e., 2000–2500 cells/spheroid) stably provided spheroids with 100–120 µm radiuses although the size of spheroids became gradually smaller due to detachment of dead cells from their surfaces. If cells were seeded at lower densities, dead cells increased, and spheroids collapsed in later stages. If cells were seeded at higher densities, nutrient/oxygen supply to inner parts of the spheroids became inadequate and cell viability reduced. Therefore, we seeded cells at the optimal densities and cultured the spheroids for eight days with half-change of medium every other day along with successive adherent culture as in our original method.

**Figure 2:**
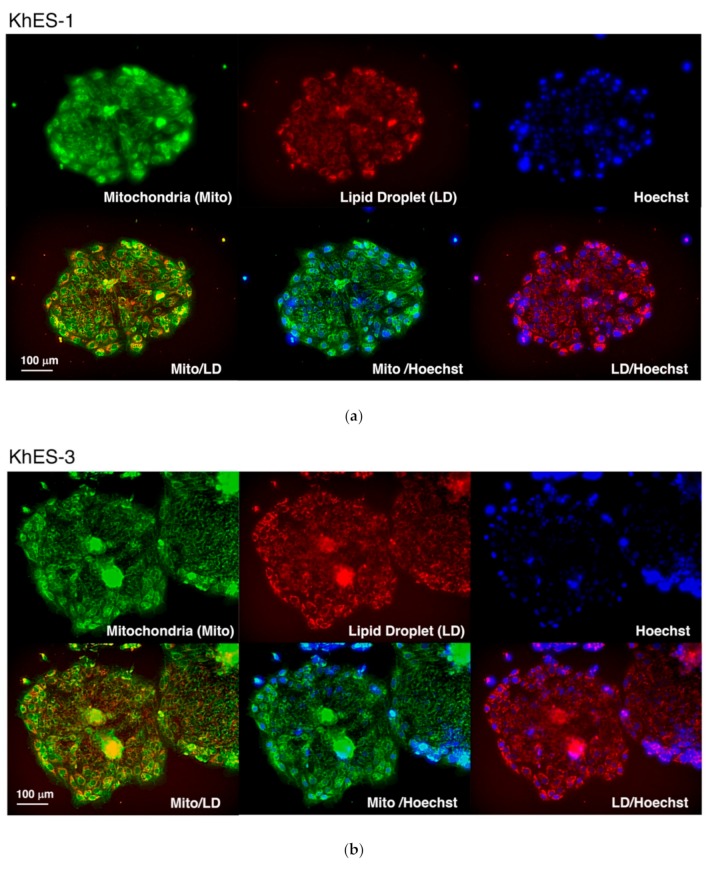

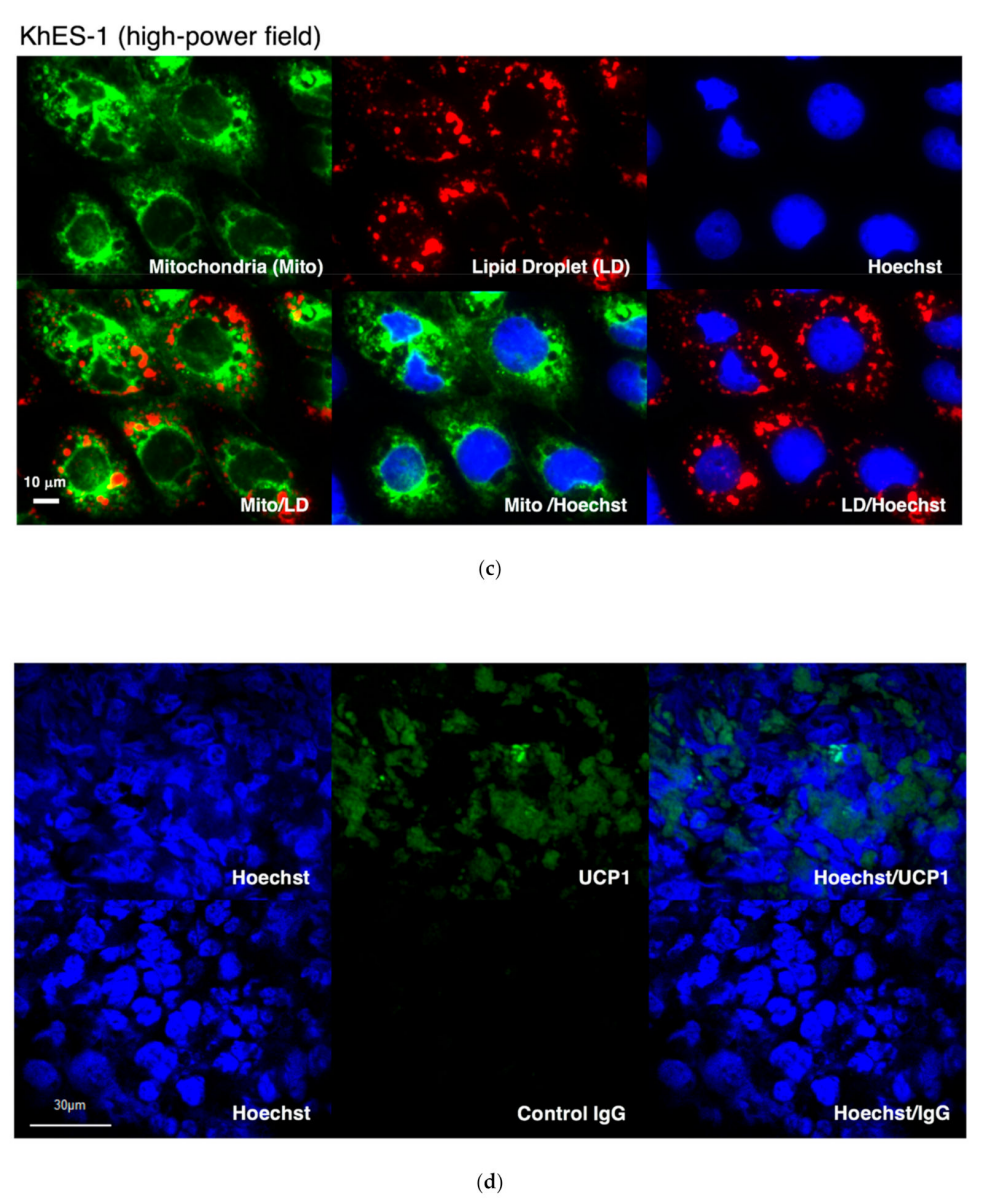
Live cell staining and immunostaining of fixed cells. The live Day 9 differentiated cells for KhES-1 (**a**) and KhES-3 (**b**) were stained by MitoBright Green (green) (Dojindo Molecular Technologies, Inc., Kumamoto, Japan), a mitochondrial probe, and Lipi-Red (red), a probe for lipid droplets, with nuclear counter staining using Hoechst 33342. High-power field images of live cell staining of KhES-1 were shown (**c**). Each cell contained abundant mitochondria and multilocular lipid droplets, which are characteristic of BA. Alternatively, the differentiated KhES-1 cells were subjected to immunostaining using an anti-UCP1 antibody (**d**).

During our preliminary trials, however, we noticed that the cells subjected to the differentiation without the cytokine cocktail showed undistinguishable morphologies from the BAs produced by our original method. To further assess this phenomenon, we tested additional cytokine-free conditions, where BSA, α-MTG, and/or PFHMII were deleted from the differentiation medium. Surprisingly, the cells subjected to the differentiation even in the absence of all these factors showed similar morphologies. We observed multilocular lipid droplets, which were detected as multiple bright round inclusions in the cytosol, and abundant mitochondria, which were detected as dark irregular particles spreading throughout the cytosol, under phase contrast microscopy (Figure 1, lower). To confirm these findings, we performed live cell staining using a probe for mitochondria (green) and a probe for lipid droplets (red). As shown in Figure 2a–c, differentiated cells uniformly showed multilocular lipid droplets along with abundant mitochondria in the cytosol, which is indicative of BA. Immunostaining using an anti-UCP1 antibody showed that the vast majority of the cells expressed UCP1 protein (Figure 2d).

**Figure 3:**
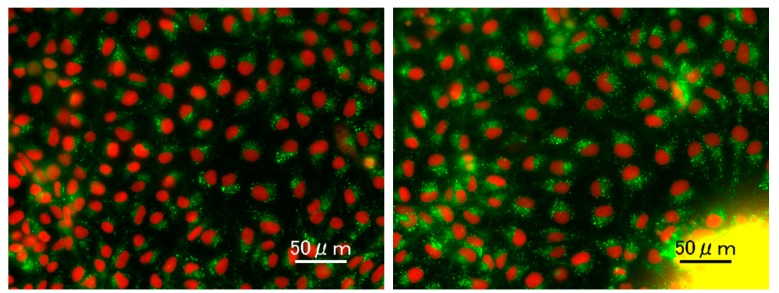
Homogeneity of the differentiation. GAPDH-2A-NLS-TdTomato knock-in KhES-3 line, whose nuclei emit red fluorescent light, was subjected to differentiation in the absence of cytokine cocktail. The presence of multilocular lipid droplets, which is characteristic to BA, was determined by live cell staining using Lipi-Green probe (Dojindo Molecular Technologies, Inc., Kumamoto, Japan).

Homogeneity of the differentiated cells was further determined by the experiments using GAPDH-2A-NLS-TdTomato knock-in KhES-3 cells, whose nuclei emitted red fluorescent light because nuclear localization signal (NLS)-fused TdTomato was expressed under the control of GAPDH by using 2A system. As shown in Figure 3a,b the differentiated cells equally showed multilocular lipid droplets in their cytosol (green), which is characteristic to BA. Moreover, their nuclei showed healthy appearance in the absence of fragmented/condensed apoptotic nuclei and swelling/melting necrotic nuclei. In the right micrograph, a yellow aggregate, which corresponded to the center of the spheroid, was seen. As in the case of Figure 2b, the spheroid was not fully expanded, and therefore, the image at this region was not clear. Nevertheless, there were no apoptotic or necrotic cells even adjacent to the yellow aggregate.

**Figure 4:**
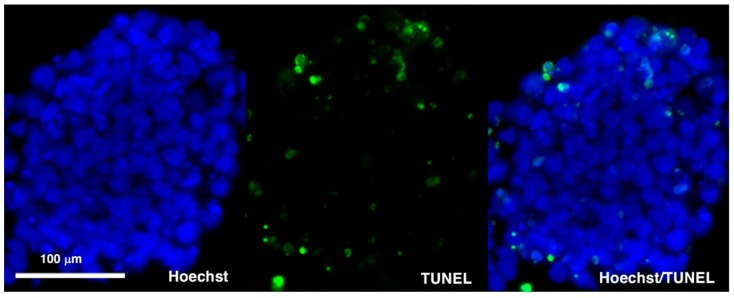
TUNEL assays. KhES-1 cells were subjected to the differentiation according to the cytokine-free method and TUNEL assays were performed using Day 10 spheroids. Representative images of Hoechst 33342 stained nuclei and TUNEL positive signals in the section of the spheroid were shown.

We also evaluated the viability of cells in spheroids. The Day 10 spheroids were fixed, sliced, and subjected to TUNEL assay to detect the apoptotic cells. Although 16.2% ± 8.8% of cells underwent apoptosis (Figure 4), there were no significant differences in the proportion of dead cells between the spheroids made by a conventional method and those made by a cytokine-free method (data not shown). Therefore, the absence of cytokine cocktail per se did not affect the viability of cells during the differentiation into BA.

**Figure 5:**
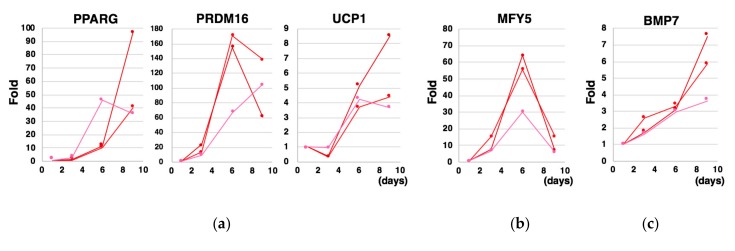
Kinetics of gene expressions during the differentiation into BA under a *cytokine-free* condition. Three independent experiments were performed. The expressions of BA marker genes (**a**), a marker for a BA progenitor (*MYF5*) (**b**), a gene that is required for BAT genesis (*BMP7*) (**c**) were examined at Day 1, Day 3, Day 6, Day 9.

We next examined the kinetics of gene expressions. We found time-dependent increments in the expressions of *PPARG*, which is known as a master transcription factor for adipogenesis, *PRDM16*, which is involved in the differentiation of MYF5-positive myoblasts into BA [6], and UCP1, which is a proton channel located at inner mitochondrial membranes and plays an indispensable role for thermogenesis through uncoupled mitochondrial respiration in response to adrenergic stimuli (Figure 5a). On the other hand, the expression of MYF-5, a marker for a BA progenitor, was transiently up-regulated, reaching a peak at Day 6 (Figure 5b). The expression of BMP7, whose knockout mice was shown to bear diminished BATs [8], was gradually upregulated during the differentiation process (Figure 5c). Interestingly, expressions of genes that code the components of cytokine cocktail were induced with its own unique kinetics (Figure 5d). Although the inductions of these endogenous cytokine genes were not always drastic, they may possibly contribute to promoting the differentiation of hESCs into BA.

**Figure 6:**
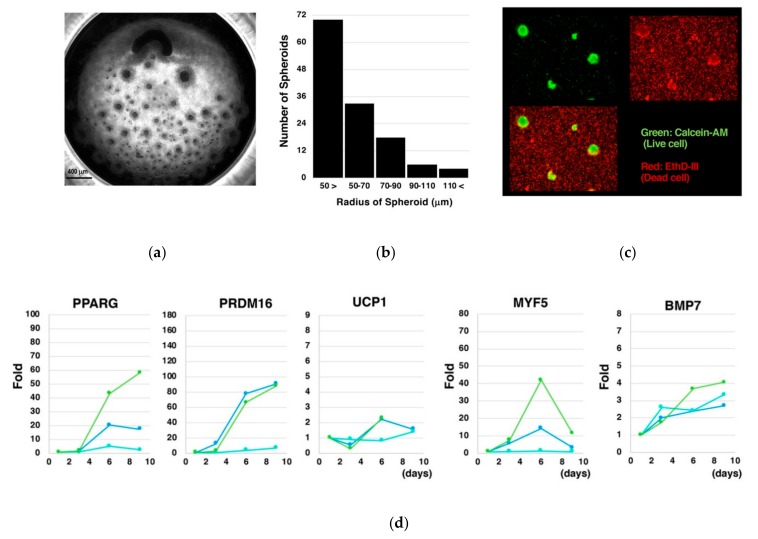
Generation of spheroids by a conventional massive floating culture lowers the efficiency of the differentiation under a cytokine-free condition. Single cell-dissociated hESCs were cultured in a 96-well flat bottom low attachment plate. Observations under phase contrast microscopy (**a**) and histogram analyses of the spheroid size (**b**) indicated that spheroids of diverse sizes with irregular shape were created. Staining with a live cell probe and a dead cell probe (**c**) showed that there were abundant dead cells which were not incorporated into spheroids. Examinations on gene expression kinetics indicated that fold inductions of the genes for BA makers (*PPARG*, *PRDM16*, *UCP1*), a myoblast marker (*MYF5*), and a BA inducer (*BMP7*) (**d**) were reduced compared to Figure 5.

To evaluate the impact of generating the size-controlled spheroids in independent microwells, dissociated cells were cultured in a 96-well flat bottom low attachment culture plate. As in our original method, cells formed spheroids with diverse sizes and irregular shapes, occasionally making huge aggregates due to lack of partitions among them (Figure 6a). The size of spheroids was small as a whole. The histogram analysis indicated that radiuses of the majority of the spheroids were smaller than 50 µm (Figure 6b). Around spheroids, there were abundant dead cells (Figure 6c). Fold inductions of the genes for BA markers (*PPARG, PRDM16*, *UCP1*), a myoblast marker (*MYF5*), and a BA inducer (*BMP7*) became lowered (Figure 6d) compared to the case where size-controlled spheroids were cultured in a mutually segregated fashion (Figure 5).

**Figure 7:**
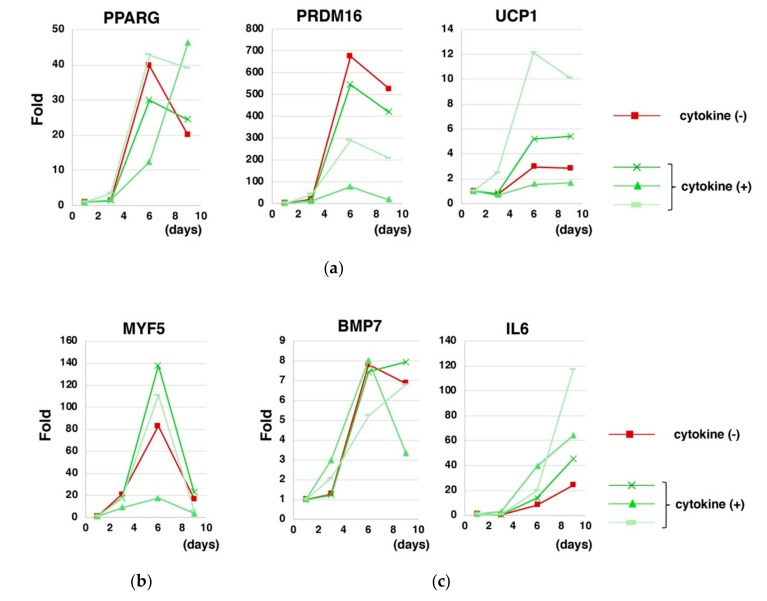
Supplementation of the cytokine cocktail did not significantly affect the differentiation of hESCs into BA in the upgraded method. hESCs (KhESC-1) were subjected to the differentiation into BA using Elplasia^®^ 3D discovery tool in the absence (red) or presence (green) of the cytokine cocktail and gene expressions were analyzed over time. The presence of the cytokine cocktail did not influence the gene expression kinetics regarding BA marker genes (**a**), BA progenitor myoblast marker gene (MYF5) (**b**), and BAT-related genes (BMP7 and IL-6) (**c**).

To confirm the dispensability of exogenous cytokines in the differentiation of hESCs using Elplasia^®^ 3D discovery tool, the cytokine cocktail was added to the cytokine-free differentiation system. We found that the presence of cytokine cocktail did not significantly affect the kinetics of gene expressions, including inductions of BA marker genes such as *PPARG*, *PRDM16*, and *UCP1* (Figure 7a), a transient induction of a myoblast marker gene *MYF5* (Figure 7b) and inductions of BAT-related genes such as *BMP7* [19] and *IL6* [3] (Figure 7c).

Those findings indicate that mechano-structural elements of the spheroids, including size (100–120 µm radiuses), regularity (orthospheres vs. aggregates), homogeneity (size-controlled vs. non-controlled), and independency (segregated vs. non-segregated), have larger impacts on the quality of differentiation than the presence of exogenous cytokines.

Collectively, hESCs can differentiate into BA via an authentic myoblastic route in the absence of exogenous cytokines if they are handled as mutually segregated size-controlled spheroids.

### 3.3. BA Produced Under an Exogenous Cytokine-Free Condition Exerts Thermogenic Activity In Vivo

**Figure 8:**
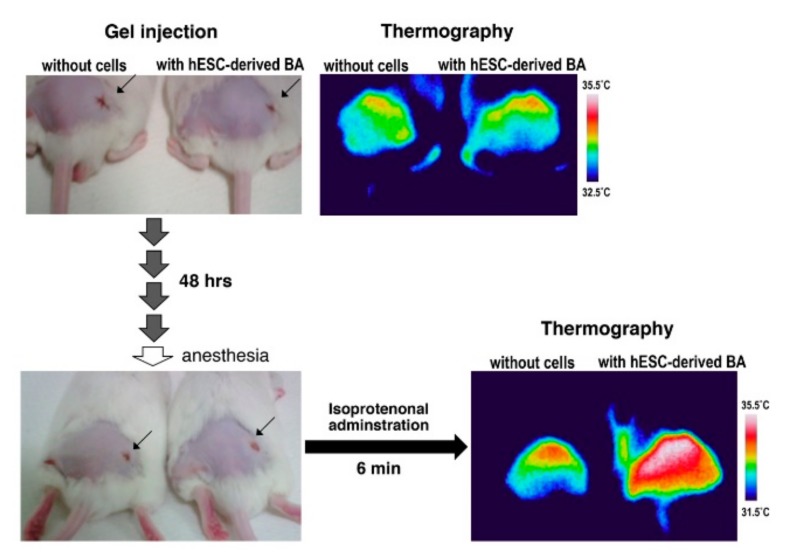
BAs generated from hESCs under cytokine-free conditions produce heat in vivo in response to adrenergic stimuli. hESCs (KhES-1) were subjected to differentiation into BA by the cytokine-free method. After being embedded in fibrin gel, BAs were subcutaneously transplanted into mice. For control, gel without cells was transplanted. After 48 h from transplantation, isoproterenol, a beta-adrenergic receptor agonist, was injected from tail vein and dermal temperature was followed up under infrared camera observations. After several minutes from isoproterenol injection, dermal temperature around the transplanted region was up-regulated specifically in BA-transplanted mice.

**Figure 9:**
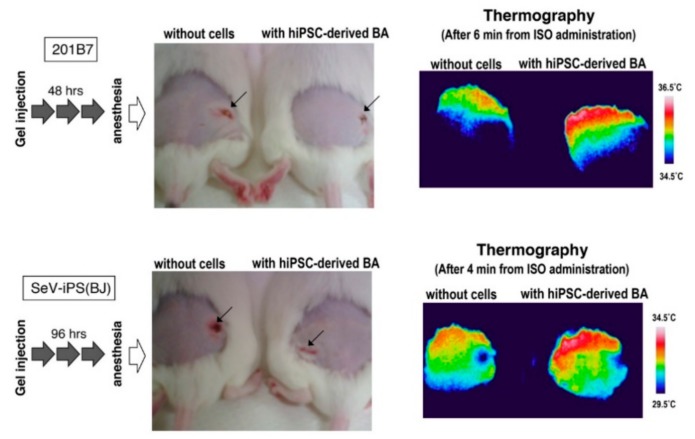
Human-induced pluripotent stem cells (hiPSC)-derived BAs produce heat in vivo in response to adrenergic stimuli. BAs were generated from two lines of hiPSCs, 201B7 (upper) and SeV-iPS(BJ) (lower) by the cytokine-free method and subcutaneously transplanted after embedded in fibrin gel. Without isoproterenol, there was no significant difference in the dermal temperature between BA-transplanted and sham-operated mice regardless of the time after transplantation. After an administration of isoproterenol, however, the dermal temperature was specifically up-regulated in BA-transplanted mice.

To evaluate functional maturation of BA produced by our cytokine-free method, the differentiated cells were subcutaneously transplanted into immunocompetent ICR mice and adrenergic receptor-dependent thermogenic activity was examined as we previously reported [4,5]. There was no significant difference in dermal temperature between empty gel-injected mice and mice transplanted with hESC-derived BA-embedded gel around the operated regions (Figure 8, upper panels). However, the dermal temperature of transplanted mice became higher than sham-operated mice after several minutes from an administration of isoproterenol (Figure 8, lower panels), indicating that BA generated by the cytokine-free method exerted thermogenic activity in vivo in response to adrenergic stimuli. Similar results were obtained from two lines of hiPSCs, 201B7 and SeV-iPS (BJ) (Figure 9).

**Figure 10:**
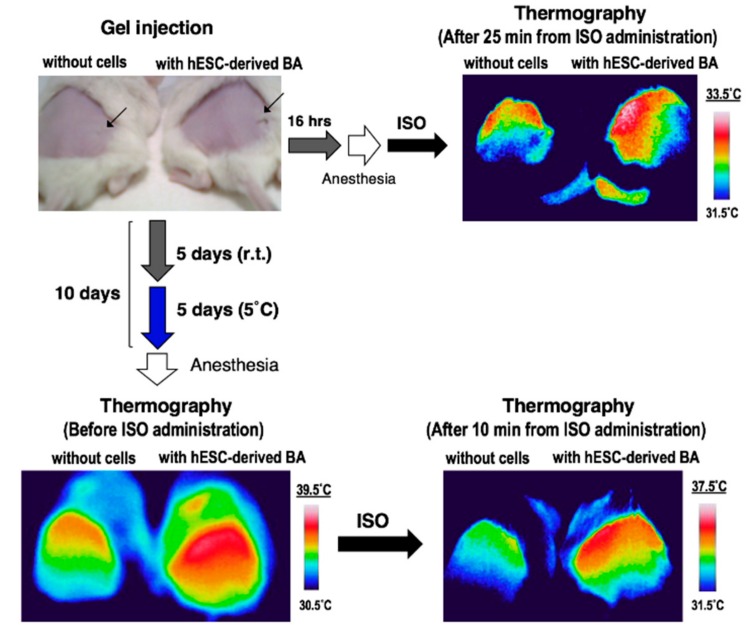
Longer-term in vivo effects of BA produced by a cytokine-free method. The KhES-1-derived BAs were transplanted into NOD/SCID mice. A total of 10 days after transplantation, 5 days in room temperature and 5 days at 5 °C, thermogenic activity was assessed.

To evaluate longer-term effects, KhES-1-derived BA was subcutaneously transplanted into immunocompromised NOD/SCID mice. We first assessed the calorigenic potential after seven days from transplantation; however, isoproterenol-dependent heat production became rather week (data not shown). Therefore, we transferred the mice into cold environments since cold acclimation is known to activate BA activity. To our surprise, mice transplanted with hESC-derived BA showed very high dermal temperature (39.5 °C) at the transplanted area after five-day acclimation even without isoproterenol administration (Figure 10, lower left). Although BA-transplanted mice showed higher dermal temperature than control mice after isoproterenol administration (Figure 10, lower right), the effect of isoproterenol per se seemed negligible when the dermal temperature of BA-transplanted mice was chased after isoproterenol administration. These findings indicate that cold acclimation is the most effective way to sustain the activity of BA in vivo as previously reported [26].

Collectively, BAs produced from human pluripotent stem cells under cytokine-free conditions are functionally maturated.

### 3.4. Partial Purification of Insulin Secretion-Enhancing Molecule from Supernatant of BA Produced Under an Exogenous Cytokine-Free Conditions

**Figure 11:**
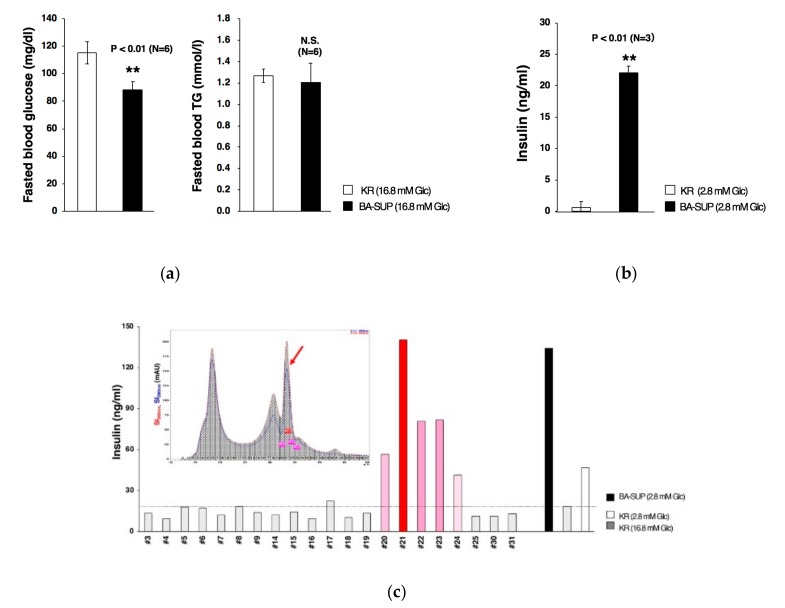
The biological effects of the supernatant of BA (BA-SUP) generated by a conventional method. (**a**) BA-SUP was prepared in the presence of high concentration glucose (16.8 mM). Control Krebs-Ringer (KR) buffer (16.8 mM) or BA-SUP was administrated to mice. After 16-h fasting, blood samples were obtained and glucose (left) and triglyceride (right) levels were measured. (**b**) BA-SUP was prepared in the presence of low concentration glucose (2.8 mM). Control KR buffer (2.8 mM) or BA-SUP was added to MIN6 cells and insulin secretion was measured after 2.5 h. (**c**) BA-SUP, which was prepared under low glucose concentration (2.8 mM), was desalted, lyophilized, and subjected to HPLC using a gel filtration column. After fractionation, each fraction was desalted, lyophilized, and added to MIN6 to assess its insulin secretion-enhancing potential. The upper panel is the graphic presentation of HPLC fractionation, where signal intensities (SI) at indicated wave lengths were followed over time. Results were reproducible and representative data were presented.

It is known that BA exerts its biological effects not only by producing heat but also by secreting physiological active substances. In our previous report, we showed that transplantation of human pluripotent stem cell-derived BA ameliorated glucose metabolism as early as 16 h after transplantation [4], suggesting that glucose metabolism improvement is not a secondary consequence of weight loss due to enhanced thermogenic activity but rather a direct effect of secreting factor(s). To evaluate this idea, we examined the biological effects of the supernatant of BA (BA-SUP) by administrating it to fasted mice. Since the differentiation medium contain high concentration glucose (17.5 mM), we prepared BA-SUP by culturing BAs, which were generated by our original method, using KR buffer supplemented with high concentration glucose (16.8 mM). Control KR buffer (16.8 mM glucose) or BA-SUP (16.8 mM glucose) was administrated to mice, and after 16-h fasting, blood samples were taken. We found that an administration of BA-SUP lowered fasted blood glucose levels (Figure 8a, left), whereas it did not affect blood triglyceride levels (Figure 11a, right). These findings suggest that, although down-regulation of blood triglyceride levels in BA-transplanted mice [4] was due to increased uptake of blood triglyceride by live BA, fasted blood glucose levels was lowered by BA-derived soluble factor(s) via enhanced insulin secretion from pancreatic beta cells. To evaluate this hypothesis, we examined the effects of BA-SUP on the basal insulin secretion activity of MIN6, a murine pancreatic beta cell line. For this aim, we prepared BA-SUP using KR buffer supplemented with low concentration glucose (2.8 mM) to minimize glucose-stimulated insulin secretion, mimicking the fasted state in vivo. We found that an addition of BA-SUP increased insulin secretion (Figure 11b), indicating that there existed a factor(s) that augmented basal insulin secretion from pancreatic beta cells. To obtain information regarding its molecular weight, we fractionated the desalted/concentrated BA-SUP by HPLC using a gel filtration column. We found that basal insulin secretion was augmented when fractions around 800 Da were added to MIN6 cells (Figure 11c, arrows). Since known insulin secretion-enhancing factors have much higher molecular weights (>3 kDa), it is strongly suggested that BA secretes a novel factor that enhances basal insulin-secreting activity.

**Figure 12:**
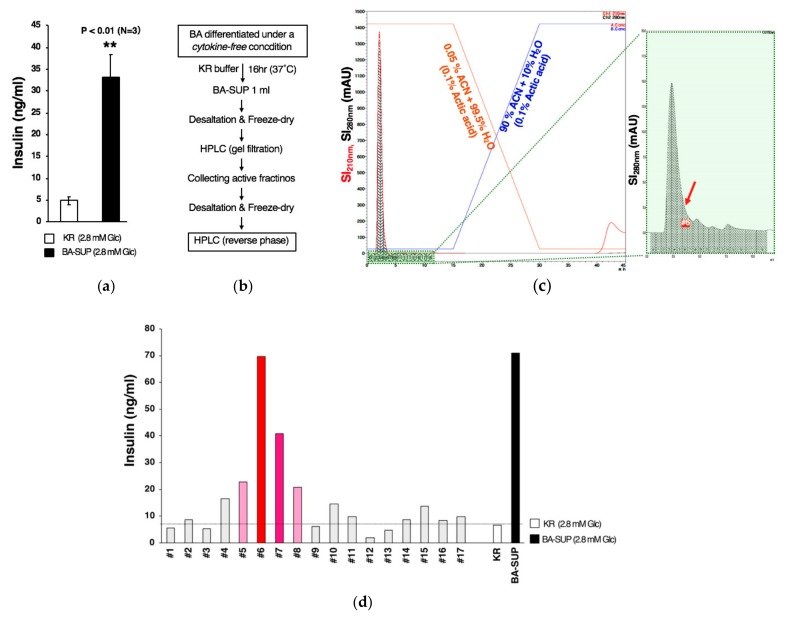
BA secretes an insulin secretion-enhancing molecule. (**a**) BAs were generated from hESCs by the cytokine-free method and BA-SUP was prepared using KR buffer containing low concentration glucose (2.8 mM). Control KR buffer (2.8 mM) or BA-SUP added to MIN6 cells and insulin secretion was measured after 2.5 h. (**b**) A schematic presentation of the partial purification of the insulin secretion-enhancing molecule. (**c**) Active fractions, which were obtained from HPLC using a gel filtration column, were mixed and subjected to reverse-phase HPLC. The results of fractionations were graphically presented by showing the signal intensities for 210 nm (red) and 280 nm (black). (**d**) The results of the insulin secretion assay using MIN6 cells were shown. The insulin secretion-enhancing activity was eluted in flow through fractions.

To exclude the possibility that the factor was a mere derivative(s) of exogenously added cytokines, we performed the experiments using the supernatant of BAs generated by the cytokine-free method. We found that an addition of BA-SUP generated under cytokine-free conditions enhanced basal insulin secretion from MIN6 cells as well (Figure 12a), confirming that the factor(s) was derived from BA. We also obtained similar results in HPLC with a gel filtration column (data not shown). Then, we collected and mixed the active fractions in gel filtration HPLC and the samples were further fractionated by reverse-phase HPLC according the procedure shown in Figure 12b. We found that the majority of the substances obtained from gel filtration HPLC was recovered in flow through fractions (Figure 12c). We further found that the activity to increase basal insulin secretion was recovered also in flow through fractions, which had been eluted before increasing the percent of Solution B (Figure 12d). We tried various kinds of hydrophobic columns; however, the active fractions were always recovered within flow through fractions, indicating that the factor is highly hydrophilic and would not bind hydrophilic resins.

Collectively, human BA secretes a certain insulin secretion-enhancing factor that has low molecular weight and is exceedingly hydrophilic.

## 4. Discussion

In the present study, we showed that human pluripotent stem cells can differentiate into BA without exogenous cytokines if cells are cultured as small homogenous spheroids (100–120 µm radiuses) in independent microwells to prevent them from fusion. We also showed that genes coding the component of the cytokine cocktail supplemented to the original differentiation medium are induced during the differentiation process with their own unique kinetics, possibly serving as auto/paracrine factors for induction and promotion of the differentiation. Although differentiated BA is subculture-incompetent as in the case of the BAs produced by our original method, our upgraded method may provide a beneficial option for the treatment of morbid obesity and obesity-related disorders. Table 1 summarizes the advantages and disadvantages in the comparison between the original and upgraded methods.

The contents of the differentiation medium used in our cytokine-free system is very simple, consisting of IMDM/Ham’s F12, synthetic lipid mixture, insulin-transferrin-selenium mixture (ITS-A), glutamine-alanine dipeptide, and ascorbic acid-2-phosphate. Although we have not yet determined the minimally essential components, our preliminary findings suggest that synthetic lipid mixture may not be requisite. We have not examined the requirement of glutamine-alanine dipeptide (GlutaMAX™ II). Nevertheless, it is widely used in in vitro culture systems as a source for glutamine, which is highly fragile under ordinary culture conditions. Therefore, the presence of glutamine-alanine dipeptide seems to be beneficial. We have not evaluated the requirement of ascorbic acid-2-phosphate either. It is known that ascorbic acid serves not only as an antioxidant, but also as a coenzyme in various reactions including lipid metabolism. For example, it plays a role as a coenzyme for the hydroxylase in biosynthesis of carnitine, which acts as a transporter of long-chain fatty acids into the mitochondria. In addition, ascorbic acid participates in cholesterol metabolism including synthesis of bile acids, which is known to increase the activity of human BAT [27]. Therefore, the presence of ascorbic acid-2-phosphate may exert a beneficial effect on the differentiation into BA. Regarding ITS-A, which is widely used in place of serum, we performed several experiments to evaluate its importance. We have found that, at least, selenium is dispensable. By contrast, depletion of transferrin considerably reduced cell viability and completely disabled the ability to form spheroids. Since the concentration of insulin in ITS-A is about 10-fold higher than human serum levels, we examined whether the concentration of insulin could be lowered. Our preliminary findings suggest that insulin concentration can be lowered, at least, to one-fifth of the ITS-A level. On the other hand, sizes of the spheroids were significantly reduced when insulin concentration was lowered to one-twentieth of the ITS-A level. Therefore, transferrin and physiological concentration of insulin are required for the differentiation into BA. Further studies are required for elucidating the minimal essential elements and their roles in inducing the differentiation.

**Figure 13:**
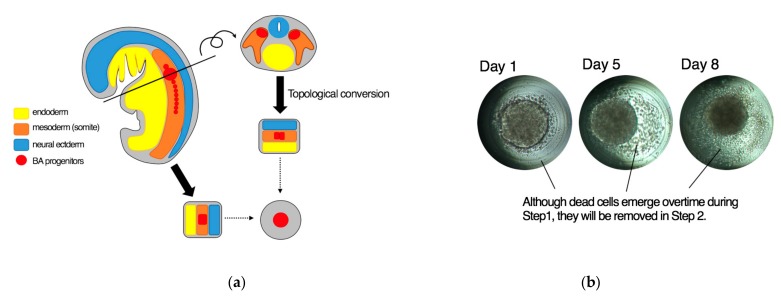
A model for the basis for the cytokine-free differentiation of human pluripotent stem cells into BA. (**a**) BA progenitors could be assumed to reside at the central portion of the developing embryo if embryos were topologically converted into spheres. (**b**) During the culture of human pluripotent stem cell-derived spheroids in a microwell under a cytokine-free condition, increasing numbers of dead cells emerge (~Day 8). However, dead cells are eliminated from the differentiation system after the start of an adherent culture at Day 8. Therefore, the central region of the spheroids consisting of live cells were preferentially transferred to the Step 2 of the differentiation system.

Although the mechanism of our cytokine-free differentiation system remains elusive, the differentiation into BA might possibly be one of the “default” capacities of human pluripotent stem cells. It has been thought that human pluripotent stem cells have a default capacity to differentiate into neuronal lineages under serum-free conditions and the presence of serum, or BMPs, affording them a capacity to differentiate into mesodermal lineages. Our results suggest that human pluripotent stem cells intrinsically have a capacity to undergo BA differentiation. BA progenitors reside a dorsal-interior area of the somite [7,28]. This area may be recognized as “the central area of the developing embryo” if embryos are topologically converted to spheres (Figure 13a). In our cytokine-free differentiation system, dead cells continuously emerge and drop off from the surface of the spheroids (Figure 13b). Therefore, the cells that have survived the differential process cells seem to be derived mainly from the central portion of the spheroids, where auto/paracrine factors were most enriched. If small size-controlled spheroids are generated and cultured in independent microwells without fusion, they would behave like the cells in the central portion of the developing body. Further in-depth studies are required to elucidate the basis of the cytokine-free differentiation of human pluripotent stem cells into BA.

Our upgraded method has a further advantage in that it affords an excellent material to explore BATokine, since it can provide BA-SUP without contamination of recombinant cytokines. As we showed in the current study, BA-SUP contains an unknown molecule that enhances insulin secretion from pancreatic beta cells. Since its molecular weight (800 Da) is significantly lower than the known molecules with insulin secretion-stimulating ability such as GLP-1 (3.3 kDa) and GIP (5 kDa), the molecule would to be a novel factor. Because it is highly hydrophilic and did not bind hydrophobic resins as far as we examined, the reversed-phase chromatography, which is of great use in peptide purification in general, is inapplicable to isolation of this novel factor from BA-SUP. Although further trials are required for its identification, our advanced system for the differentiation of human pluripotent stem cells may contribute to the discovery of novel substances that will not otherwise be identified. The discovery of such novel factors will promote an advancement of personalized medicine. Currently, there are only a few genes whose polymorphisms are shown to be associated with BAT activities. For example, polymorphisms of the genes involved in thermogenesis such as *UCP1* and *ADRB3* are shown to be associated with age-related changes in human BAT [29]. However, it is known that BAT contributes to metabolism improvement via secreting factors as well (i.e., BATokines). Unfortunately, genuine BATokines, which should be specifically produced by BA, have not yet been identified primarily due to lack of appropriate research tools. Our system, which provides an ideal tool for BATokine exploration, will open the door to BAT-focused personalized medicine for the diagnosis and treatment of metabolism disorders such as obesity and type 2 diabetes.

## 5. Conclusions

hESC/hiPSC spontaneously differentiate into BA without exogenous cytokines if they are cultured as size-controlled spheroids (100–120 µm radius) in independent microwells with half-change of medium every other day.

## 6. Patents

The original method for a directed differentiation of human pluripotent stem cells into brown adipocytes is internationally patented (Japan 5998405; United States 9492485; Australia 2012248333; China ZL201280020534.X; EU granted on 12 June 2018, currently under registration procedure in Switzerland, Germany, France, and United Kingdom).

A method for preparing biologically active culture supernatant from brown adipocytes generated from human pluripotent stem cells under a cytokine-free condition is currently Japanese patent pending (Tokugan 2018-152727, Tokugan 2019-026463).

A method of purification and the identification of the molecule with basal insulin secretion-stimulating activity is currently Japanese patent pending (Tokugan 2019-12945).

## Figures and Tables

**Table 1:** Comparison between the two methods.

	Original Method	Up-Graded Method
Culture plate	any low attachment plates will do	plates that guarantee size-controlled spheroid formation
Cytokine cocktail	required	dispensable
Routine medium-changing procedure	easy and simple	requires labor and skill
Number of cells that can be handled at a time	large	small

## Supplementary Materials

The following are available online at https://www.mdpi.com/2073-4409/8/4/373/s1, Figure S1: A directed differentiation of feeder-free hESCs into classical BA using 96-well V-shaped plates.
