# Supplementary material for: Exogenous Cytokine-Free Differentiation of Human Pluripotent Stem Cells into Classical Brown Adipocytes
Source: Cells. 2019 Apr 24;8(4):373. doi: 10.3390/cells8040373 (PMC6523334; doi:10.3390/cells8040373)
Supplement: Supplementary file 1 [file cells-08-00373-s001.pdf]

Supplementary Materials

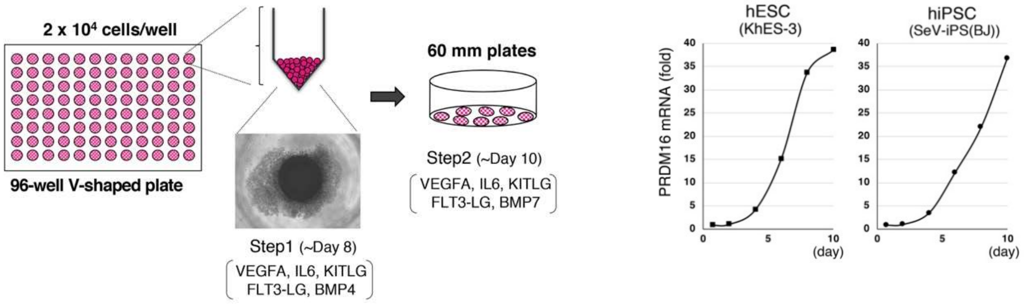

(**a**)

(**b**)

Figure S1. A directed differentiation of feeder-free hESCs into classical BA using 96-well v-shaped plates. (**a**) Schematic presentation of the procedure. hESCs and hiPSCs, which were maintained using StemFitTM AK02N (Ajinomoto Healthy Supply Co.) on vitronectin-coated plates, were dissociated into single cell in the presence of a ROCK inhibitor and subjected to spheroid formation by seeding in a low attachment V-shaped 96-well plate (PrimeSurfaceTM 96V well culture plate). Cells were cultured for eight days using the differentiation medium supplemented by a cytokine cocktail with half-change of medium every other day, followed by adherent culture for successive two days in 60 mm gelatin-coated plates using the differentiation medium supplemented by another cytokine cocktail as previously described. (**b**) hESC and hiPSC were collected at indicated time after an induction of differentiation. PRDM16 mRNA expressions were examined by RT-qPCR.
